# Intrinsic signalling factors associated with cancer cell-cell fusion

**DOI:** 10.1186/s12964-023-01085-5

**Published:** 2023-04-04

**Authors:** Thomas Dittmar, Ralf Hass

**Affiliations:** 1 Institute of Immunology, Centre for Biomedical Education and Research (ZBAF), Witten/Herdecke University, Stockumer Str. 10, 58448 Witten, Germany; 2 Biochemistry and Tumor Biology Laboratory, Department of Obstetrics and Gynaecology, Hannover Medical School, 30625 Hannover, Germany

**Keywords:** Cancer cell fusion, Fusogens, Post-hybrid selection process (PHSP), Phosphatidylserine (PS), Pro-fusogenic state, Pre-hybrid preparation program (PHPP)

## Abstract

Cellular fusion e.g. between cancer cells and normal cells represents a stepwise process that is tightly regulated. During a pre-hybrid preparation program somatic cells and/or cancer cells are promoted to a pro-fusogenic state as a prerequisite to prepare a fusion process. A pro-fusogenic state requires significant changes including restructure of the cytoskeleton, e.g., by the formation of F-actin. Moreover, distinct plasma membrane lipids such as phosphatidylserine play an important role during cell fusion. In addition, the expression of distinct fusogenic factors such as syncytins and corresponding receptors are of fundamental importance to enable cellular mergers. Subsequent hybrid formation and fusion are followed by a post-hybrid selection process. Fusion among normal cells is important and often required during organismal development. Cancer cells fusion appears more rarely and is associated with the generation of new cancer hybrid cell populations. These cancer hybrid cells contribute to an elevated tumour plasticity by altered metastatic behaviour, changes in therapeutic and apoptotic responses, and even in the formation of cancer stem/ initiating cells. While many parts within this multi-step cascade are still poorly understood, this review article predominantly focusses on the intracellular necessities for fusion among cancer cells or with other cell populations of the tumour microenvironment.

Video Abstract

**Supplementary Information:**

The online version contains supplementary material available at 10.1186/s12964-023-01085-5.

## Introduction

The hypothesis that cancer cells can fuse with other cells, such as macrophages, mesenchymal stroma-/stem-like cells (MSC), or further populations, with evolving cancer hybrids of an altered phenotype, was already postulated by the German physician Otto Aichel more than a century ago [1]. Such altered cancer hybrids can exhibit an enhanced metastatic capacity, an increased drug resistance and prospective cancer stem/initiating cells (CS/ICs) properties as described in a plethora of in vitro and in vivo studies (for review see: [2–8]). While these studies indicate that cancer cells and other cells could fuse, it not only remains unclear how this process is directed in detail, but also *why* cancer cells, or at least a certain subpopulation could *fuse at all*.

The fusion of cancer cells represents a pathophysiological process with increased malignancy that can ultimately be life-threatening to the organism. With respect to a variety of different tumour entities it is well known that development of metastases are the main cause of death in more than 90% of cases [9, 10]. Accordingly, cancer cell fusion, e.g. with MSC can develop cancer hybrid cells from a subsequent post-hybrid selection process (PHSP) with significantly increased metastatic capacity [11, 12].

Cancer hybrid cells may also contribute to immune escape [6, 13–22]. Gast et al. and Powell et al. suggested that leukocyte properties alone were sufficient to explain why (circulating) tumour × leukocyte hybrids may exhibit an immune-privileged phenotype [16, 17]. YKL-40 and immune checkpoint protein B7-2 (CD86) were elevated in glioblastoma × macrophage hybrids and independently suppressed anti-tumour immune factor levels of CD8^+^ cytotoxic T lymphocytes resulting in escape of immune surveillance [14]. Aguirre and colleagues observed that in vitro-derived cancer hybrid cells expressed high levels of the immune checkpoint ligand PD-L1, which was correlated to a reduced CD8^+^ lymphocyte proliferation in a PD-L1/PD-1 interaction-dependent manner [22]. Similarly, NK cell activity was significantly downregulated due to higher expression of NK cell inhibitors HLA class I members [22]. Likewise, HERV *env* proteins, such as syncytin-1 and syncytin-2, themselves exhibit immune suppressive properties, whereby it remains to be clarified how these proteins suppress host immunity [19, 20]. Syncytin-1 is commonly associated with (cancer) cell-cell fusion (Table 1) [23–33], but exhibits further tumour promoting characteristics, such as proliferation, invasion, metastasis [26, 34, 35], and possibly even immune escape. However, the precise role of cancer hybrids in immune escape still remains to be clarified. A possible focus could be on syncytins and checkpoint inhibitors.

**Table 1:** Syncytin-1 expression in cancer

Cancer type	Fusogen/ cell	Fusogen receptor/ cells	Effect on cancer progression	References
Breast cancer (cells)	Syncytin-1/ Breast cancer cells	ASCT2/ HUVEC	Cell fusion was correlated with improved prognosis	[23, 24]
	SyHP/ Breast cancer cells	Not analysed	PGCC formation	[29]
Breast epithelial cells	Syncytin-1/ Syncytin-2/ Breast cancer cells	ASCT2/ MSFD2A/ MSC	Not investigated	[121]
Colon cancer cells	Syncytin-1, CD9, CD47/ Colon cancer cells	Not analysed	PGCC formation	[27]
	Syncytin-1/ Colon cancer cells	Not analysed	Overall disease progression	[32]
Endometrial cancer (cells)	Syncytin-1/ Endometrial cancer cells	Not analysed	Cell-cell fusion; Proliferation; invasion	[26, 35]
NSCLC	Syncytin-1/ NSCLC cells	Not known/ NSCLC cells	Overall disease progression	[31]
OSCC cells	Syncytin-1/ OSCC cells	ASCT2/ HUVEC	Cell-cell fusion	[30]
prostate cancer (cells)	Syncytin-1/ Prostate cancer cells	Not known/ Skeletal/ smooth muscle cells	Cell-cell fusion	[28]
seminomas	Syncytin-1/ Seminoma cells	Not analysed	Not analysed	[33]
UCC	Syncytin-1/ UCC	Not known/ UCC	Cell-cell fusion; Proliferation	[25]

In contrast to cancer cell fusion, physiological fusion processes, such as fertilization, placentation and myogenesis, are essential for an organism. Proper functioning of these fusion processes are ultimately required since dysregulation and/or dysfunction are associated with infertility and embryonic lethality [36–43]. Tissue regeneration represents another physiological cell-cell fusion process, which is facilitated by MSC and bone marrow-derived cells (BMDCs) [17, 44–57]. Even though the potency of BMDC-mediated tissue regeneration via cell-cell fusion was demonstrated in several in vivo studies [17, 44–57], the overall involvement of BMDCs in maintaining tissue homeostasis and repair remains less clear.

While all cell-cell fusion processes converge at a shared pathway of phospholipid bilayer fusion, the merger of cancer cells as compared to normal cells such as trophoblasts (placentation) and myoblasts (myogenesis) must be different processes. In addition to the fundamental difference on the physiology of the organism, this also includes the induction and regulation of cell-cell fusion. In particular, placentation and myogenesis include tightly regulated and very efficient fusion processes giving rise to a high number of viable multinucleated syncytial cells [42, 43, 58, 59]. In contrast, the fusion of cancer cells either with further cancer cells or with other cell populations like macrophages or MSC reveals a low fusion rate. Moreover, the overall survival of the resulting cancer hybrids is extremely low due to the reorganization of aneuploid chromosomal nuclei in a subsequent PHSP.

Accordingly, cancer cell fusion is influenced by both intrinsic signals and extracellular events. These questions will be explored and addressed in this review, with a particular focus on intrinsic signalling factors.

## Similarities between physiological and patho-physiological cell-cell fusion events with a focus on fusogens, membrane phospholipids, and the actin cytoskeleton

**Fig. 1:**
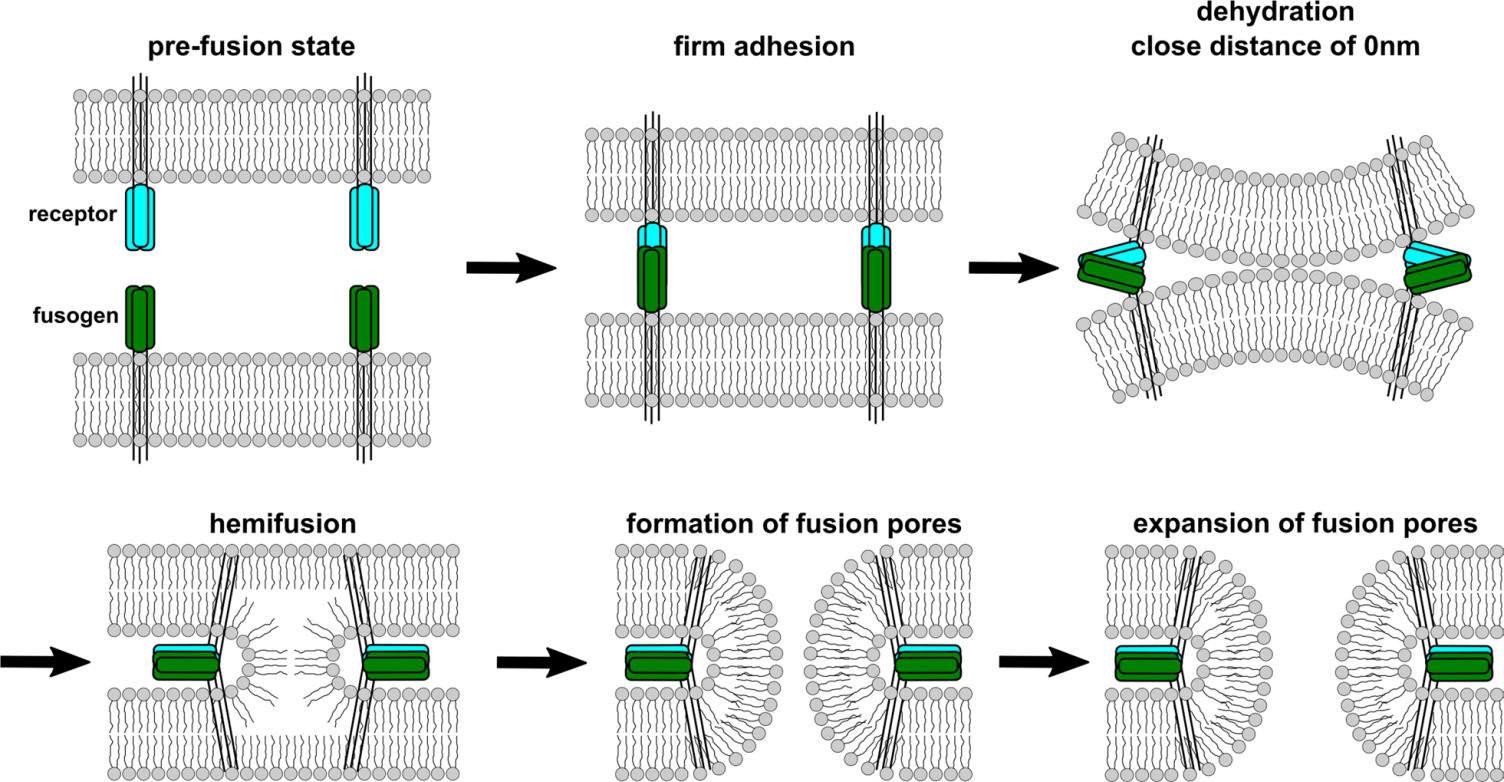
Schematic scheme of decisive steps in membrane fusion. Fusogens ultimately catalyse the merger two phospholipid double membranes that should actually repel each other due to their negative charge

The merger of bilayered phospholipid membranes of vesicles (e.g. endo-and exocytosis), organelles (e.g. mitochondria, exosomes), infection of host cells with enveloped viruses, or cell-cell merger represent fusion processes. These are complex, multi-factorial (including various proteins, phospholipids and biophysical conditions), energy depending, tightly and timely regulated but still scarcely understood actions (for review see: [3–5, 40, 60–69]). Figure 1 illustrates the apparently conserved pathway of lipid rearrangements in fusion of plasma membranes of cancer cells and normal cells, e.g. trophoblasts (placentation) and myoblasts (myogenesis) (Fig. 1).

In addition to other proteins/ factors, a decisive role in cell-cell fusion is displayed by distinct proteins and phospholipids, which are indispensable for plasma membrane merger. These include so-called fusogens and their cognate receptors, as well as phosphatidylserine (PS), scramblase activity and PS-binding proteins [60–62, 64–67].

### Fusogens and cognate receptors during physiological and non-physiological cell-cell fusion events

Although fusogens share the similar functionality to promote membrane merger, they differ in tissue-specific structure and compartmentalization and thus, display a marked heterogeneity. Syncytin-1 (human endogenous retrovirus-type W (HERV-W)) and syncytin-2 (human endogenous retrovirus-type FRD (HERV-FRD)) represent fusogens, which belong to the large family of HERV elements that account for about 8% of the human genome. Syncytin-1 and -2 are structurally related to class I viral fusogens [60, 61, 66, 70–72]. In a physiological environment these fusogens facilitate the fusion of villous and extravillous cytotrophoblasts to form syncytiotrophoblasts during placentation [73–78]. In the course of placenta formation Sugimoto and colleagues demonstrated that the human endogenous retroviral (HERV) element suppressyn impaired syncytin-1-mediated cytotrophoblast fusion by binding to the syncytin-1 receptor alanine, serine, cysteine transporter 2 (ASCT2) [79]. Moreover, increased suppressyn serum levels were found in women with placental defects in Down syndrome pregnancies [80]. In fact, a disturbed or dysregulated trophoblast syncytialization during placentation is commonly associated with an impaired maintenance and failed integrity of the blood-placental barrier. These effects could lead to pregnancy disorders with subsequent development of pre-eclampsia, HELLP syndrome, intrauterine growth retardation and even miscarriage [41, 79–84]. These unwanted complications and potential outcomes underline the importance of syncytin-1 functionality and proper cell-cell fusion during placental development.

In addition to placentation, syncytin-1 might also be involved in osteoclastogenesis [85, 86]. Here, data of Moller et al. revealed that syncytin-1 rather promoted cell fusions of two multi-nucleated osteoclasts, whereas the fusion of mono-nucleated pre-osteoclasts is directed by CD47 [85]. These findings suggest that different fusogens are involved in promoting and regulating either mono-nucleated or multi-nucleated cell fusion of the same cell type.

Other cell-type specific fusogens include the single pass transmembrane protein myomerger (myomixer—minion) and the seven transmembrane protein myomaker by promoting myoblast fusion [38–40]. A peak expression of both myomaker and myomerger is observed during skeletal muscle development [38]. Little if any multinucleated muscle cells were found in myomaker-myomerger knockout embryos [58, 87, 88]. This was further correlated with a lack of skeletal muscle wrapping, a transparent appearance and an overall early embryonic lethality [58, 87, 88].

At the level of gametes representing haploid sets of chromosomes, Juno and Izumo1 are important mediators of oocyte and sperm fusion [36, 37]. While Juno is a folate receptor (also known as folate receptor 4), Izumo1 represents a transmembrane protein in sperms. The ectodomain is composed of an immunoglobulin-like domain. The Izumo domain can be further divided into the N-terminal unstructured region and an α-helical core region important for sperm–egg binding [37]. Likewise, Juno and the tetraspanin-4 family protein CD9 may be important factors in oocytes. Accordingly, knockout of either Izumo1 (sperm), or Juno or CD9 (both oocyte) was associated with an impaired cell-cell fusion ability and, hence, correlated with infertility [37, 89, 90]. Recent work suggested that, in addition to its role in sperm to egg binding, Izumo1 exhibits fusogen activity [91]. Together, these data indicate the necessity of functioning fusogens and subsequent proper cell-cell fusion in various physiological processes and during development of an organism.

A different picture is observed in a patho-physiological environment such as the fusion of cancer cells. In contrast to the multiple fusogens that are involved in a variety of different physiological cell fusion events, only syncytin-1 has been identified with cancer cell-cell fusion and tumour progression so far [23–33, 35]. Interestingly, a potential association of syncytin-1 with cancer cell fusion was documented in various different tumour entities as summarised in Table 1.

It still remains unclear why syncytin-1 is expressed by cancer cells. Several studies have demonstrated an intrinsic basal syncytin-1 expression in various cancer cells and tumour types, which could be caused by changes in the promoter region and/ or other regulatory elements, such as 3’-long-term-repeat (LTR) and 5’-LTR regions [23–33]. Similarly, other data provided evidence that cytokines and an oestrogen responsive element, respectively, could induce, and thus increase intrinsic syncytin-1 expression levels in cancer cells and different tumour entities [23, 24, 26–30, 32]. Additionally, viral infections could also induce syncytin-1 expression in different cell types, such as Epstein-Barr virus (EBV) and human immunodeficiency virus (HIV) in astrocytes and monocytes [92, 93], and SARS-CoV-2 in Calu-3 cells and A459-ACE2 lung cancer cells [94]. Although these data indicate a possible link between certain viral infections and the induction of syncytin-1 expression, it is completely unclear if this actually plays a role in cancer cell fusion. Nevertheless, the tropism of viruses must also be taken into account, since viruses can only specifically infect their host cells.

Together, previous studies demonstrated that cancer cell-cell fusion is mediated primarily by syncytin-1, which appears similar to physiological syncytin-1-mediated cell-cell fusion events. However, no other fusogens have been identified so far in cancer cell fusion. In this context, studies by Uygur et al. showed that PC3 prostate cancer cells can fuse with muscle cells. In contrast to the muscle-specific fusogens myomaker and myomerger, however, this fusion process was also mediated by syncytin-1 [28].

### Phosphatidyl serine (PS) in normal and cancer cell fusion

The membrane phospholipid component PS has been suggested as a uniquely conserved molecule in cell-cell and virus-cell fusion [62]. Under normal conditions PS is localised in the inner leaflet of the plasma membrane whereby translocation to the outer leaflet can be associated with apoptosis [95]. However, this PS shuttling is also essential for cell fusion. The two Ca^2+^-activated phospholipid scramblases (Ca^2+^-PLS) TMEM16E and TMEM16F, have been associated with cell-cell fusion [84, 96–99]. In particular, TMEM16F facilitates the translocation of PS from the inner to the outer leaflet of the plasma membrane which is essential for trophoblast fusion. Conversely, TMEM16F knockout mice exhibited a deficiency in trophoblast syncytialization and placental development, which was correlated to perinatal lethality [84].

Likewise, muscle progenitor cells from adult TMEM16E^−/−^ knockout mice exhibited defective cell-cell fusion in culture and produced muscle fibres with significantly fewer nuclei as compared to controls [96]. This markedly reduced fusogenic capacity was associated with a decreased Ca^2+^-dependent PS exposure on the surface of TMEM16E^−/−^ muscle progenitor cells and a decreased Ca^2+^ amplitude [96]. Re-expression of TMEM16E fully restored the fusogenic capacity of muscle progenitor cells concomitant with PS translocation and normalised Ca^2+^-dependent currents [96]. In opposite to these findings, however, studies by Gyobu and colleagues reported no apparent skeletal muscle abnormalities in TMEM16E^−/−^ mice [97]. These discrepancies were hypothesised to be related to genetic differences in the murine animal models, to different approaches for down-modulation of TMEM16E, or to other experimental factors [96].

Fertilization represents another PS-dependent mechanism. Viable sperms containing membrane-associated PS and corresponding PS recognition receptors on oocytes have been suggested as key players in sperm × oocyte fusion [99]. Indeed, fertilization was impaired by both, masking of PS or functional disruption of PS receptor-specific signal transduction pathways [99]. Similarly, fertility was impaired in TMEM16E^−/−^ mice, which, in addition to impaired PS translocation, was also attributed to a reduced sperm motility [97].

While the above summarised studies indicate the importance of PS in plasma membrane merger of normal cells, the role of PS in cancer cell-cell fusion is less clear. Only two studies so far suggested the involvement of PS in cancer cell-cell fusion [28, 100]. Muscle cell-mediated increase in IL-4 and IL-13 levels induced syncytin-1 and annexin A5 expression in human PC3 prostate cancer cells and subsequent cancer cell-cell fusion [28]. By contrast, hybridization of these two cell types was impaired by annexin A5 knockdown [28], which supports the necessity of PS even in cancer cell-cell fusion.

Whereas PS translocation to the outer plasma membrane leaflet can be associated with either apoptosis or cell fusion Noubissi et al. reported that the fusion between MSC and T47D human breast cancer cells or between MSC and MCF7 human breast cancer cells was significantly enhanced by apoptosis [100]. These findings suggest that apoptosis-induced cancer cell-cell fusion could result in viable cancer hybrids with a mixed genotype. Moreover, early apoptotic cells with still intact genome may represent the preferred fusion partners. Simultaneously, engaged and running pro-apoptotic pathways must be terminated in these newly formed cancer hybrids. Hochreiter-Hufford et al. demonstrated that apoptosis together with induced signalling via the PS receptor BAI1 promoted myoblast fusion [101]. Notably, apoptotic cells were only in contact with viable fusing myoblasts/myotubes, but did not merge with them indicating that cell-cell fusion was attributed to BAI1-induced signalling [101]. It is quite conceivable that a similar mechanism may occur in apoptosis-mediated fusion of cancer cells.

In brief, there is growing evidence for the importance of PS in plasma membrane-membrane fusion of normal cells and cancer cells [62, 66]. Nonetheless, the regulation of Ca^2+^-PLS activation and subsequent PS shuttling within the plasma membrane remains unclear although different Ca^2+^-PLS regulation pathways/ mechanisms have been identified [62, 66]. Moreover, potential signalling cascades in PS-relayed apoptosis and/or cell fusion represent crucial mosaic pieces within the multifactorial cell-cell-fusion machinery.

### Actin in cell fusion

In addition to PS translocation and the expression of certain fusogens, restructure of certain cellular compartments including the actin cytoskeletal system is further required for cell-cell fusion [102, 103]. Thus, actin polymerization and associated cytoskeletal proteins play a substantial role to provide a fusion-permissive structure for the fusogenic cellular partners.

Changes in intracellular contractile elements of cytoskeletal proteins and a switch between a more soft and a rigid trabecular system of actin/myosin components promotes firm adhesion, higher migratory capacity, and facilitates cellular interactions. This is supported by the activation of the small GTPases Rac1 and Cdc42, and the Arp2/3-WASP complex which are key effectors of the actin cytoskeleton protrusion machinery to reorganise the actin cytoskeleton and promote an actin-mediated cell motility [104–107]. Interestingly, many genes required for myoblast fusion have been identified in fly, zebrafish and mouse, which have in common that they are participating in Arp2/3-mediated actin polymerization and formation of podosome-like structures [64, 108]. In fact, before the discovery of myomerger and myomixer, the formations of podosomes and invadopodia were thought to be instrumental in myoblast fusion. Reorganization and accumulation of F-actin results in the formation of F-actin-based protrusions that penetrate the target cell, causing the formation of fusion pores with ultimate fusion of the cells [108].

It is well-known that the Arp2/3 complex is deregulated in various tumours. Overexpression of Arp2/3-WASP is tightly associated with markedly enhanced cancer cell invasion and disease progression of several tumour types, including breast, lung, colorectal, prostate, and pancreas (for review see: [109]). The impact of Arp2/3-WASP in cancer cell-cell fusion is unclear, but previous work suggested a substantial role of F-actin polymerization and associated cytoskeletal protein alignment to enable a permissive microenvironment for the fusion of breast cancer cells with MSC within the tumour microenvironment [110, 111]. This fusion process could be inhibited by cytochalasin D which blocks elongation of actin filaments but exhibited no detectable effects on the expression of integrins and various cell adhesion molecules. Likewise, latrunculin B which belongs to a family of macrolide-structured toxins prevented the complex formation of monomeric G-actin with ATP and thereby impaired the energy-dependent generation of F-actin structures [110]. These findings suggest that cytoskeletal components including F-actin play an important role to provide a pro-fusogenic intracellular structure by interacting with distinct lipids of the cell membrane.

**Fig. 2:**
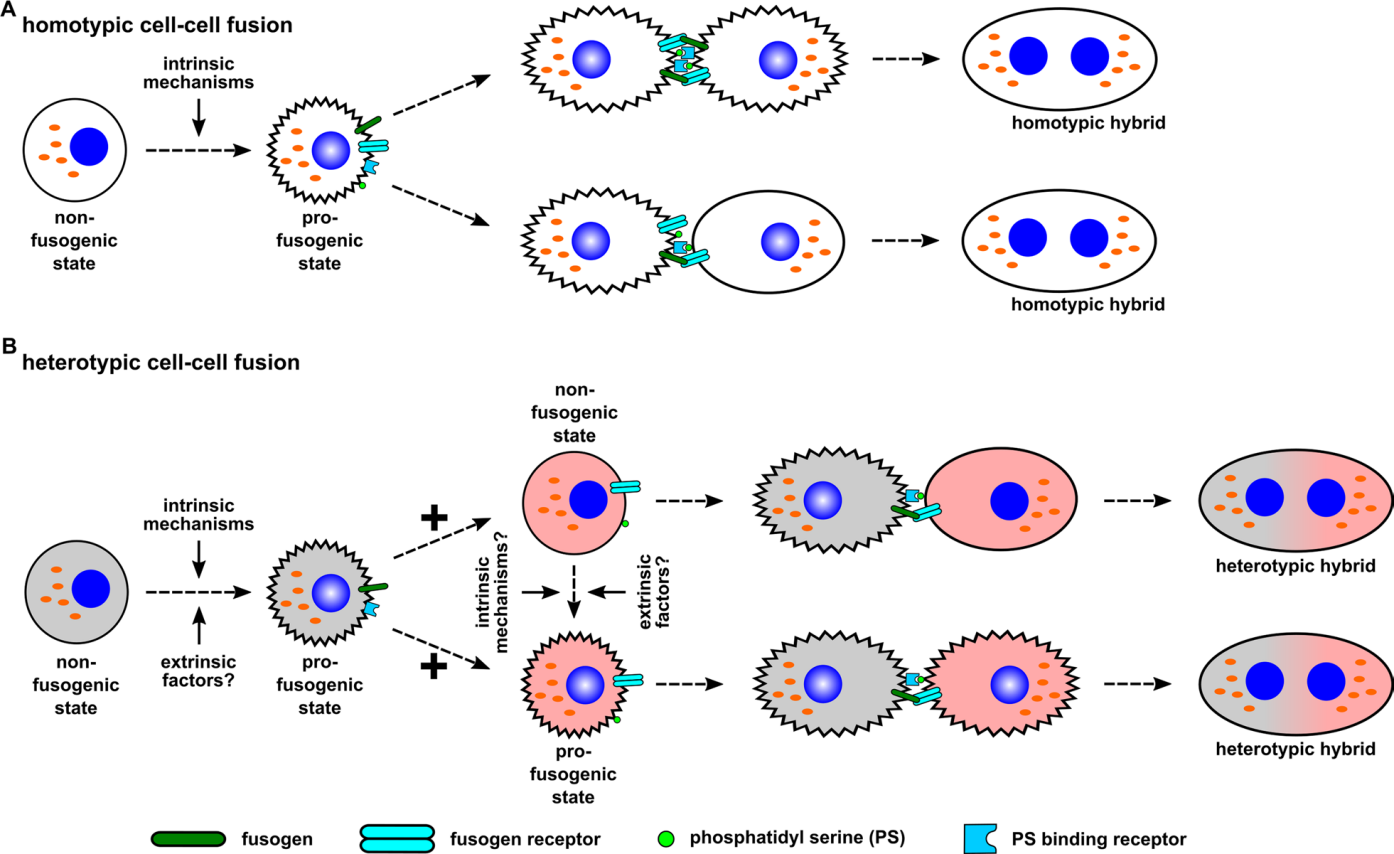
Homotypic and heterotypic cell-cell fusion. Physiological homotypic cell-cell fusion is characterised by a highly efficient fusion frequency and the generation of multiple syncytia, which is most likely attributed to a high number of cells in a pro-fusogenic state (**A**). However, it is also possible that pro-fusogenic cells fuse with non-fusogenic cells in a homotypic manner. Heterotypic cell-cell fusion is different since it remains unclear, which cells provide fusogens and exhibit further fusion relevant properties (**B**). A pro-fusogenic cell fuses with a cell in a non-fusogenic state, which only expresses cognate receptors and phosphatidylserine (PS) (adapted from [166])

Taken together, cells are non-fusogenic per se, but must first undergo a pre-hybrid preparation program (PHPP) to promote transition into a so-called hypothetical pro-fusogenic state [4, 5, 60, 61, 112]. Fertilization, placentation, and myogenesis represent physiological cell-cell fusion processes that rely on important intrinsic signalling mechanisms. Structural cytoskeletal requirements in concert with sequential and timely availability of fusogens, corresponding fusogen receptors, and further proteins/phospholipids involved in fusion mediate transition into a pro-fusogenic state for preparation of cell fusion. However, cancer cells actually represent a non-fusogenic cell population although some studies indicated that cancer cells intrinsically can expressed fusogens such as syncytin-1 [23–33, 35]. A summary of these different models is presented in Fig. 2.

Thus, some basic components of cell-cell fusion in physiological and pathophysiological processes may not differ from each other with respect to the involved proteins/phospholipids. Nevertheless, there are significant differences at the regulatory levels.

## Differences between physiological and non-physiological cell-cell fusion events

The fundamental differences between physiological and pathophysiological cell-cell fusion events are their impact on the organism. Physiological fusion processes are essential for an organismal development whereby dysregulation and/or dysfunctions are associated e.g. with infertility and embryonic lethality [36–43]. In contrast, the pathophysiological process of cancer cell fusion is associated with a significantly enhanced tumour plasticity [12, 113]. Usually, this leads to disease progression due to an increased malignancy of the tumour hybrids [2–8]. Physiological and pathophysiological cell-cell fusion events unravel further differences, including the general fusion frequency, the regulation of the fusion process itself, the fusion partner, and the survival rate of the hybrid cells after a PHSP.

### Regulation of cell-cell fusion and general fusion frequency

Fertilization, placentation, and myogenesis represent examples for physiological cell-cell fusion events that are characterised by an inherent time-dependent expression of specific fusogens and cognate receptors [3–5, 40, 60–69]. All of these fusion processes are running in a controlled manner and are terminated at a certain time point to avoid additional unwanted cell-cell fusion events. In particular, placentation and myogenesis represent highly effective cell-cell fusion events, which give rise to numerous syncytia in vitro and in vivo. For example, immortalised murine C2C12 myoblasts fused at a similar rate as compared to human skeletal myoblasts [59]. A lot of fusion events were also observed between nuclei present in cultured syncytia and fibroblasts expressing both, myomaker and myomerger [58]. Likewise, marked levels of myotube formation were determined by Isobe and colleagues using a novel fusion quantification system [42]. In this line, primary human trophoblasts cultured for 72 h and BeWo cells treated with forskolin for 48 h demonstrated a significant amount of fusion event [43]. These high cell-cell fusion rates during placentation and myogenesis are achieved by a precisely timed expression/activation of the cell-cell fusion machinery. For instance, a continuous syncytin-1 expression is detectable in villous cytotrophoblasts until 37 weeks of gestation [41]. Conversely, a peak expression of both, myomerger and myomerger is observed during skeletal muscle development [38].

In contrast, the fusion of cancer cells is different since only a certain subpopulation of cancer cells exhibits fusogenic properties. Moreover, the fusion frequency can change markedly between distinct cancer cell lines and read-out systems. Accordingly, determination of fusogenecity in vitro varied between 0.0066 and 6.5% [114–118] and in vivo between 0.5 and 51% [16, 17, 57, 115, 118–120]. However, the in vivo data have to be regarded very carefully as they do not necessarily represent the actual fusion frequency of the (cancer) cells. This particularly applies to detection limitations during in vivo studies with different tumour models, different readout systems, and especially different time points applied.

These data demonstrate that cancer cells apparently have an inherent fusogenecity that appears to be different among various cancer cell lines and only applies to a certain subpopulation. Moreover, the overall fusogenecity of cancer cells/ cell lines is rather low when compared to the rates observed during physiological cell-cell fusion events.

### Cancer cell fusion partners: homotypic versus heterotypic fusion

Cell-cell fusion can be divided into homotypic and heterotypic processes. In homotypic cell-cell fusion events identical cell types are merging. Conversely, during heterotypic fusion hybrids are formed with different cell populations. Homotypic cell fusion predominantly applies to physiological processes, e.g. placentation and myogenesis (Fig. 2A) [3–5, 40, 60–69]. As an exception, heterotypic physiological cell-cell fusion is represented by the sperm and oocyte fusion with the rearrangement of haploid sets of chromosomes during fertilization [3–5, 40, 60–69].

Cancer cells may fuse in a homotypic as well as in a heterotypic manner [2–8]. According to the hypothesis of a pro-fusogenic state as a prerequisite for cell-cell fusion, either one or both cancer cells should acquire this state (Fig. 2A, B). A similar picture would emerge for heterotypic cancer cell fusion events (Fig. 2B). Either both or one of the cancer cell or normal cell would be in a pro-fusogenic state (Fig. 2B). In addition, a direct fusion between a pro-fusogenic cancer cell and a pro-fusogenic normal cell is also possible. This may account for a higher fusion rate in heterotypic cancer cell fusion since two independent pro-fusogenic cell types are involved. Supportive evidence for this model was obtained from previous work which determined a significantly lower homofusion (autofusion) rate by more than tenfold in various breast cancer cell lines when compared to a corresponding heterofusion with MSC [121].

**Fig. 3:**
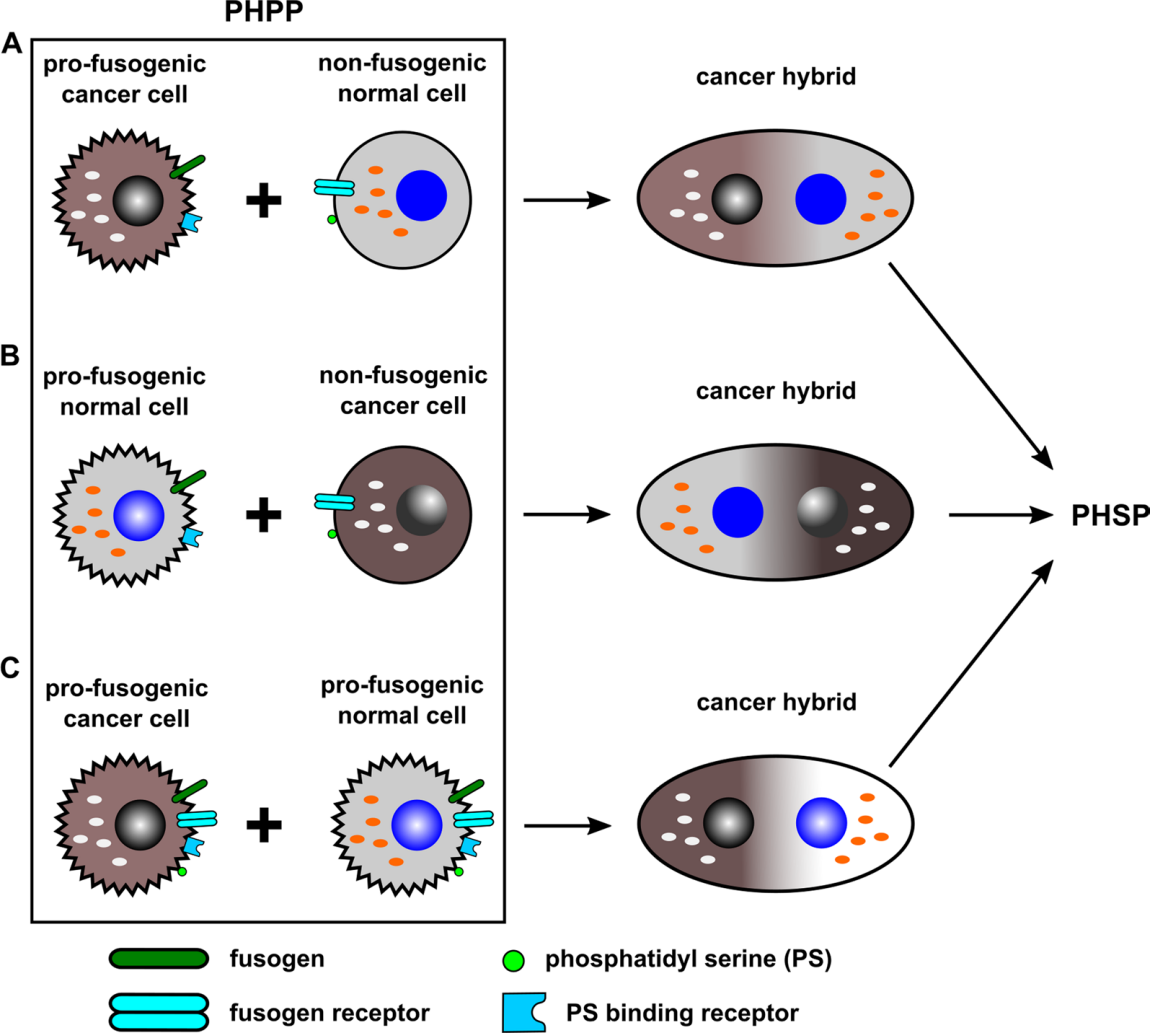
In this model it is assumed that cancer cells fuse with other cells suggesting that all cells have entered a pre-hybrid preparation program (PHPP) by acquisition of a pro-fusogenic state (**A**). Alternatively, it cannot be ruled out that other cells in a pro-fusogenic state, such as macrophages, MSC, or stem cells could fuse with cancer cells in a non-fusogenic state (**B**). Similarly, both a cancer cell and a normal cell must have acquired a pro-fusogenic state for fusion (**C**). The resulting cancer hybrid populations from all of these alternatives have to undergo a post-hybrid selection process (PHSP) for re-organization of the chromosomal ploidy and corresponding metabolic adaptation to ensure survival

As described above, expression of syncytin-1 in cancer cells appears to be primarily involved in cell fusion [23–33, 35]. These findings fit well to the assumption that “cancer cells fuse with other cells” (for review see [3–5, 61, 63, 122, 123]), which implies that cancer cells have acquired a pro-fusogenic state, express all necessary components of the fusion machinery and then actively fuse with other non-fusogenic cells (Fig. 3A).

In addition, normal cells such as macrophages and MSC also exhibit fusogenic properties by acquisition of a pro-fusogenic state to form hybrids with other cells including cancer cells (Fig. 3B) [121, 124–130]. This assumption resembles MSC- and BMDC-mediated tissue regeneration e.g. during tumour growth-mediated chronic inflammation [17, 44–57, 131–135]. Tumour invading MSC and macrophages do not distinguish between normal cells and cancer cells and exhibit their regenerative capacity according to the requirements of the tissue damage [136, 137].

### Heterokaryon-to-synkaryon transition/ ploidy reduction and post hybrid-selection process

Cell-cell fusion in neoplastic tissues represents a rare process. But even rarer is the survival of the resulting hybrid cells. The fusion of two cells does not simply result in the duplication of the karyotype. Rather, in case of division-active cells, such as cancer hybrids, two complex processes follow, which are the heterokaryon-to-synkaryon transition/ploidy reduction [135, 138–140] and PHSP [5]. Both processes are associated with aneuploidy and genomic instability due to chromosome missegregation, DNA damage, micronuclei formation and chromothripsis [141–146]. Thus, not only the fusion process itself, but also the survival probability of tumour hybrids in general is rather rare. Nonetheless, previous work assumed that despite a relatively low fusion frequency, fusion-mediated recombination could have a profound impact on clonal diversity and an overall increased intratumoural heterogeneity [113, 115].

This raises the question if cancer hybrid cells following HST/PR and PHSP are generally associated with increased malignancy? Besides increased malignancy, different outcomes have been described including reduced tumourigenicity [126, 147, 148] and tumour dormancy [149]. It is conceivable that HST/PR- and PSHP-associated cellular and genotypic stresses are survived primarily by those cancer hybrids in which apoptosis pathways are turned off and survival signalling pathways are active. Such cancer hybrids could exhibit a selection advantage over chemotherapeutic agents. Nonetheless, the fact that survival of fused cancer hybrid cells represents a rare event reflects one of the major differences to physiological cell-cell fusion processes.

## Conclusion

Beside the importance of physiological cell fusion, there is profound scientific consensus that cell-cell fusion events also occur in human cancers and that disease progression can be related to the formation of tumour hybrids [16, 124, 150–158]. However, precise regulatory mechanisms to control e.g. fusion of mononucleated versus multinucleated cells are still obscure.

**Fig. 4:**
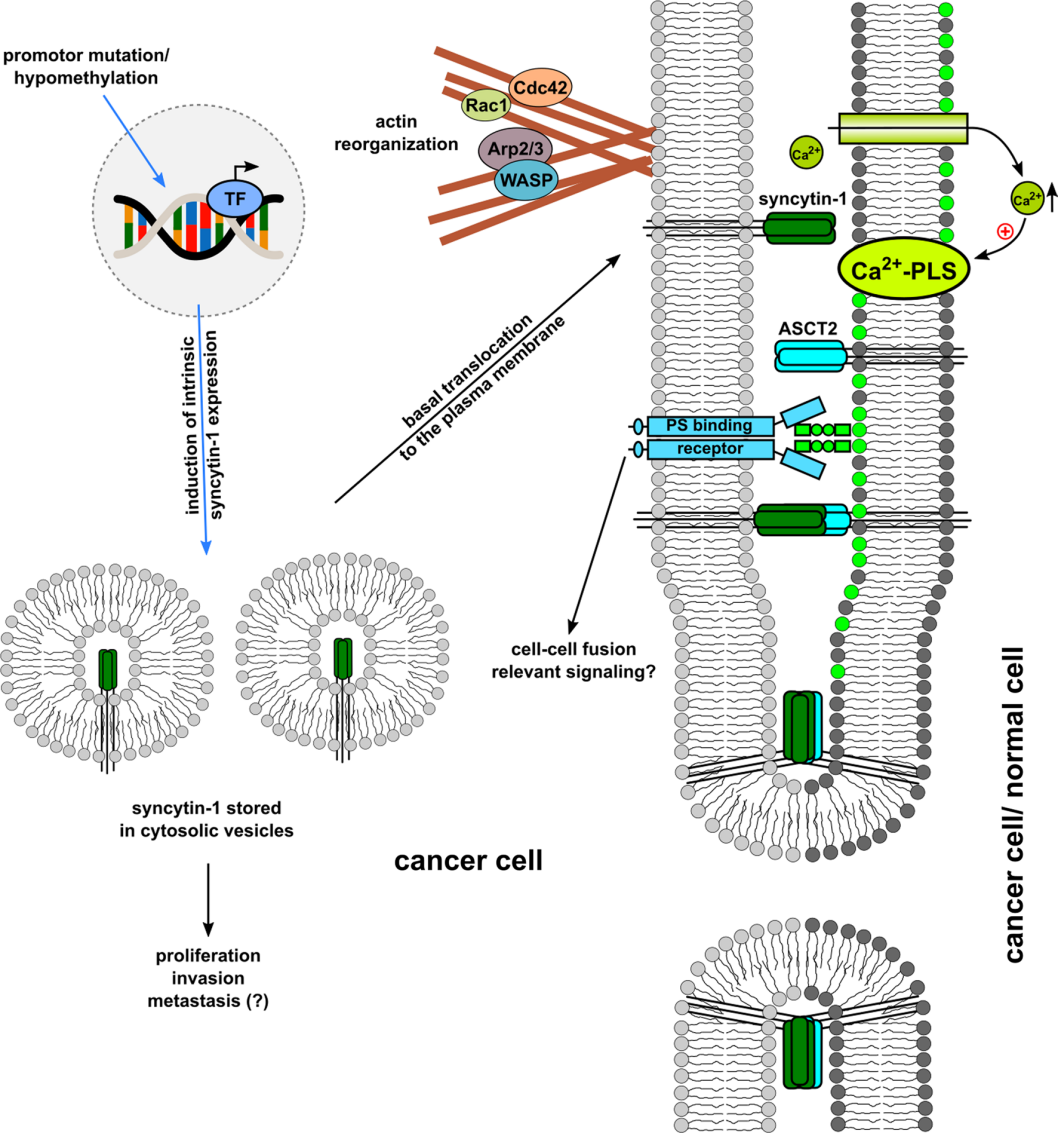
Cancer cell-cell fusion model which is focused predominantly on the expression of the fusogen syncytin-1 and includes some basal similarities to physiological fusion events. In this model it is assumed that a cancer cell with a re-organised actin cytoskeleton expresses syncytin-1 and a PS-binding receptor, which recognises ASCT2 and PS, respectively. A basal syncytin-1 expression could be attributed to promotor mutation and/or hypomethylation (TF = transcription factor). The majority of expressed syncytin-1 will remain in the cytosol. In some cells, syncytin-1 may translocate to the plasma membrane, which may be facilitated by altered cytoskeletal structures. The corresponding cell exhibits Ca^2+^-PLS activity, which is engaged by Ca^2+^ to shuttle PS and subsequently enable plasma membrane merger and cell fusion (modified according to [166])

Although some mechanistic principles of cell-cell fusion do not markedly differ between physiological and pathophysiological processes (Fig. 4), other fundamental differences can be observed. These include, for example, the high fusion frequency in physiological processes such as placentation and myogenesis, which is ensured by a time-controlled upregulation of the fusion machinery. In contrast, fusion of cancer cells represents a rare process that is predominantly relayed by a basal expression of the fusogen syncytin-1. The rarity of cancer cell fusion events is also based on the limitation of fusogens expressed only in certain cancer subpopulations. In this context, the significantly higher rates of cancer cell heterofusions (e.g. with MSC) as compared to homofusions [121] are explained by the availability of more fusogenic partner cells and support the hypothetical fusion models (Figs. 2 and 3). In addition to the low cancer cell fusion rates, the number of surviving tumour hybrids is even far lower as a consequence of restoring DNA stability during the PHSP [5]. This is also consistent with mathematical models which show that despite a relatively low fusion frequency, fusion-mediated recombination could have a profound impact on clonal diversity and an overall increased intratumoural heterogeneity [115].

If the amount of PHSP-surviving aneuploid cancer hybrid cells is obviously negligibly low why care for these cells? Previous work has demonstrated in vitro and in vivo that certain cancer hybrid cells with aneuploid karyotype, e.g. after fusion of breast cancer cells with MSC acquire a proliferation advantage during the selection process beside other properties such as enhanced metastatic capacities. Consequently, the cancer hybrid cells rapidly overgrow the initial cancer cells by exhibition of new properties [11, 119] and thus, expanding the tumour heterogeneity and plasticity. Of interest, a summarising compilation revealed various tumour-derived aneuploid cell lines. These include among others different carcinoma types and tissues such as 12 × breast, 4 × ovarian, 4 × cervical, 4 × endometrial, 4 × brain, 4 × lung, 7 × colon, 4 × liver, 5 × kidney, 5 × pancreas, 4 × gastric, 3 × prostate, beside melanoma, osteosarcoma, retinoblastoma, nasal, pharyngeal soft palate cancer, and 4 × leukaemia [159]. Although no direct proof is available, most of these spontaneously tumourigenic patient-derived cancer cells may be associated with previous fusion events after surviving from a PHSP as aneuploid cell types. Nevertheless, other forms of cancer cell mergers such as cannibalism or entosis can also contribute to aneuploid outcomes [160, 161] and even a more metastatic phenotype [162]. However, these findings demonstrate that a negligibly low amount of cancer hybrid cells can have a significant impact on the corresponding tumour development after all.

It still remains unclear why syncytin-1 is expressed only in a specific fraction of cancer cells. Previous work has demonstrated that proliferation, invasion and potentially metastasis of cancer cells may be influenced by syncytin-1 [26, 34, 35]. Similar findings have already been described for other HERV *env* elements [25, 163–165] indicating also a possible non-fusogenic role of syncytin-1 in cancer progression.

The timely orchestration of intrinsic signalling pathways involves actin-mediated intracellular restructuring to enable cytosolic syncytin-1 transport to the plasma membrane for a pro-fusogenic state. Mechanisms that facilitate this syncytin-1 translocation are unclear. In addition, Ca^2+^-PLS, particularly TMEM16F facilitates PS shuttling from the inner to the outer leaflet of the plasma membrane as an essential prerequisite for membrane merger. A variety of further components such as e.g. extracellular events [166] conclude a PHPP for subsequent cell fusion.

Interestingly, the fusogenic properties of macrophages and MSC are rather underestimated in the context of cancer cell-cell fusion. This is surprising, since many studies have shown that these cells can regenerate tissue damage through cell-cell fusion [17, 44–57]. Tumour invading macrophages and MSC seem to ignore as to whether normal cells or cancer cells represent their interaction partner. Moreover, the tumour microenvironment resembles chronic inflammatory tissue [131–133] which attracts macrophages and MSC and is a known inducer of cell-cell fusion [51, 56, 167].

It is therefore important to unravel the detailed interplay of the fusogenic contributors and their regulation particularly at the molecular level of the entire cell fusion process. Further insights into the underlying mechanisms could help to develop selective anti-cancer cell fusion strategies in tumour therapeutic approaches.

## Data Availability

The datasets used and/or analysed during the current study are available from the corresponding author on reasonable request.

## Abbreviations

ASCT2: Alanine, serine, cysteine transporter 2
EBV: Epstein-Barr virus
BMDCs: Bone marrow-derived cells
Ca^2+^-PLS: Ca^2+^-activated phospholipid scramblases
CS/ICs: Cancer stem/initiating cells
HERV: Human endogenous retroviral
HERV-FRD: Human endogenous retrovirus-type FRD
HER2-W: Human endogenous retrovirus-type W
HUVEC: Human umbilical vein endothelial cells
HIV: Human immunodeficiency virus
LTR: Long-term-repeat
MSC: Mesenchymal stroma-/stem-like cells
NSCLC: Non-small cell lung carcinoma
OSCC: Oral squamous carcinoma cells
PGCC: Polygiant cancer cells
PHPP: Pre-hybrid preparation program
PHSP: Post-hybrid selection process
PS: Phosphatidylserine
SARS-CoV-2: Severe acute respiratory syndrome corona virus-2
syHP: Syncytin-1 homologous protein
TMEM16: Transmembrane member 16
UCC: Urothelial cell carcinoma
